# Generation of a SARS-CoV-2-susceptible mouse model using adenovirus vector expressing human angiotensin-converting enzyme 2 driven by an elongation factor 1α promoter with leftward orientation

**DOI:** 10.3389/fimmu.2024.1440314

**Published:** 2024-12-09

**Authors:** Yusuke Matsumoto, Tomoko Honda, Fumihiko Yasui, Akinori Endo, Takahiro Sanada, Sakiko Toyama, Asako Takagi, Tsubasa Munakata, Risa Kono, Kenzaburo Yamaji, Naoki Yamamoto, Yasushi Saeki, Michinori Kohara

**Affiliations:** ^1^ Department of Microbiology and Cell Biology, Tokyo Metropolitan Institute of Medical Science, Tokyo, Japan; ^2^ Transboundary Animal Diseases Research Center, Joint Faculty of Veterinary Medicine, Kagoshima University, Kagoshima, Japan; ^3^ Protein Metabolism Project, Tokyo Metropolitan Institute of Medical Science, Tokyo, Japan

**Keywords:** COVID-19, SARS-CoV-2, mouse model, adenoviral vector, proteomics

## Abstract

**Introduction:**

To analyze the molecular pathogenesis of severe acute respiratory syndrome coronavirus-2 (SARS-CoV-2), a small animal model such as mice is needed: human angiotensin converting enzyme 2 (hACE2), the receptor of SARS-CoV-2, needs to be expressed in the respiratory tract of mice.

**Methods:**

We conferred SARS-CoV-2 susceptibility in mice by using an adenoviral vector expressing hACE2 driven by an elongation factor 1α (EF1α) promoter with a leftward orientation.

**Results:**

In this model, severe pneumonia like human COVID-19 was observed in SARS-CoV-2-infected mice, which was confirmed by dramatic infiltration of inflammatory cells in the lung with efficient viral replication. An early circulating strain of SARS-CoV-2 caused the most severe weight loss when compared to SARS-CoV-2 variants such as Alpha, Beta and Gamma, although histopathological findings, viral replication, and cytokine expression characteristics were comparable

**Discussion:**

We found that a distinct proteome of an early circulating strain infected lung characterized by elevated complement activation and blood coagulation, which were mild in other variants, can contribute to disease severity. Unraveling the specificity of early circulating SARS-CoV-2 strains is important in elucidating the origin of the pandemic.

## Introduction

Severe acute respiratory syndrome coronavirus-2 (SARS-CoV-2) is causing a worldwide pandemic. Development of animal models that recapitulate coronavirus disease 2019 (COVID-19) is essential for evaluating vaccines and antivirals, and for understanding the pathogenesis of the disease. Although several SARS-CoV-2 animal models have been described to date, such as monkeys, ferrets, and hamsters (1–6), mice are considered to be better suited for use as animal models given their small size, rapid breeding cycles, and well-characterized immunological background. However, due to the lack of the viral entry receptor, human angiotensin converting enzyme 2 (hACE2), laboratory mouse strains are non-permissive to most circulating SARS-CoV-2 infections (7–9). Several groups have reported mouse models that either permanently or transiently express hACE2. There are three transgenic mouse models using different promoters to drive permanent hACE2 expression, including a universal cytomegalovirus (CMV) enhancer/beta-actin promoter (10), an epithelial cell-specific promoter (K-18 or HFH4) (11, 12) and the endogenous mouse ACE2 promoter (13). Nevertheless, the production of hACE2 transgenic mice is time-consuming and their versatility is limited as they are restricted to a single genetic background. Temporary hACE2 expression in mice can be achieved rather quickly by adeno-associated virus (AAV)- or adenovirus type 5 (Ad5)-mediated transduction. SARS-CoV-2 replication in mice sensitized by AAV-hACE2 transduction appears to be lower than that reported in other mouse models (14). Transduction of mice with CMV promoter-driven hACE2-expressing Ad5 confers SARS-CoV-2 susceptibility, but only mild to moderate pulmonary pathogenesis has been induced by SARS-CoV-2 infection (15, 16). The induction of an antiviral immune response to the adenovirus vector itself is supposed to have a negative effect on SARS-CoV-2 infection, which may inhibit severe disease in the mouse respiratory tract (17). The commonly used CMV-driven transgene-expressing Ad5 vectors lack the intrinsic viral E1 gene that is essential for adenovirus growth, thereby allowing the insertion of a transgene. When the expression unit was inserted in the rightward orientation, a viral pIX gene located downstream of the inserted unit was co-expressed with the transgene, and a fusion protein consisting of the N-terminal part of the transgene product was expressed. These pIX products may be one of the main causes of adenovirus-induced immune responses. Interestingly, the EF1α promoter did not activate the pIX promoter in this adenoviral vector (17). The EF1α promoter with a leftward orientation resulted in a reduced antiviral response and maintained prolonged transgene expression (17).

In this study, we established a novel COVID-19 mouse model using an adenoviral vector expressing hACE2 under the EF1α promoter with a leftward orientation for the evaluation of the molecular pathogenesis of SARS-CoV-2 infection in the respiratory tract *in vivo*.

## Materials and methods

### Ethics statement

All experiments using mice were approved by the Tokyo Metropolitan Institute of Medical Science Animal Experiment Committee and were performed in accordance with the animal experimentation guidelines of the Tokyo Metropolitan Institute of Medical Science.

### Cells and viruses

Vero E6/TMPRSS2 cells, which constitutively express human TMPRSS2 (18), and human embryonic kidney 293 (HEK293) cells were maintained in Dulbecco’s modified Eagle’s medium (DMEM) supplemented with 10% fetal bovine serum (FBS), penicillin and streptomycin, and G-418 (1 mg/mL, only in VeroE6/TMPRSS2 cells). All cells were cultured at 37°C in 5% CO_2_. SARS-CoV-2 early circulating strain (TY/WK-521, GISAID ID: EPI_ISL_408667) and **v**ariants of concern [QHN001 (B.1.1.7 Alpha strain, GISAID ID: EPI_ISL_804007), TY7-501 (P.1, Gamma strain, GISAID ID: EPI_ISL_833366) and TY8-612 (B.1.351, Beta strain, GISAID ID: EPI_ISL_1123289)], which were all broadly used in previous reports (19–21), were obtained from the National Institute of Infectious Diseases, Japan (passage 1). All studies used passage 2 of SARS-CoV-2 that was generated in Vero E6/TMPRSS2 cells by infecting viruses with passage 1 at an MOI of 0.001. Cell culture media was harvested at 2 to 3 dpi. The sequence of the S gene of all stock viruses was confirmed to be similar to those annotated in the GISAID database. All replication-competent SARS-CoV-2 experiments were performed in a biosafety level-3 (BSL-3) laboratory in the Tokyo Metropolitan Institute of Medical Science.

### Generation of rAd5 pEF1α-hACE2-L

E1- and E3-deleted adenovirus derived from human adenovirus type 5 encoding expression units with a leftward orientation were used in this study, as described previously (17, 22). The hACE2 gene was cloned in the antisense orientation into pAxCAwtit2, the adenoviral cosmid vector that contains the left end of adenovirus type 5 with the E1 region substituted with an expression cassette containing the EF1α promoter and a multicloning site using an Adenovirus Dual Expression Kit (Takara Bio, Tokyo, Japan). The rAd5 pEF1α-hACE2-L was generated by transfecting pAxCAwtit2 encoding hACE2 into HEK293 cells by using a CalPhos Mammalian Transfection Kit (Takara Bio). The rAd5 pEF1α-hACE2-L was purified using two rounds of cesium chloride gradient centrifugation, and the titers of the concentrated and purified virus stocks were determined using HEK293 cells and an Adeno-X Rapid Titer Kit (Takara Bio) according to the manufacturer’s instructions.

### Plaque formation assay

Vero E6/TMPRSS2 cells in six-well plates were washed with DMEM-GlutaMAX, inoculated with serially diluted SARS-CoV-2, and incubated at 37°C for 60 min with rocking every 15 min. After removing the viruses, cells were washed with DMEM-GlutaMAX and overlaid with agarose medium. After incubation of cells at 37°C for 2 days, the plaques were visualized with crystal violet staining and counted.

### Mouse study

Specific pathogen-free 7-8-week-old female BALB/c CrSLC mice were purchased from Japan SLC (Hamamatsu, Japan) and housed in a temperature-controlled BSL-3 animal facility in the Tokyo Metropolitan Institute of Medical Science. All of the animals were given free access to food and water and were maintained on a 12 h light/12 h dark cycle.

Mice were intranasally inoculated with 1, 2.5, or 5 × 10^7^ focus forming units (FFU) rAd5 pEF1α-hACE2-L/mouse in a 50 µL volume. Five days after inoculation, SARS-CoV-2 was intranasally inoculated in a 50 µL volume. Prior to inoculation, mice were anesthetized by intraperitoneal administration of ketamine-xylazine mixture. Mice were euthanized by exsanguination under isoflurane anesthesia before autopsy for tissue collection.

### Viral RNA quantification

The left lung lobe from each mouse was homogenized in nine volumes of Leibovitz’s L-15 medium (Thermo Fisher Scientific, Waltham, MA, USA) using a Multi-Bead Shocker (Yasui Kikai, Osaka, Japan). Total RNA samples were extracted from 50 μL of the supernatant of lung homogenates using Isogen LS (Nippon Gene, Tokyo, Japan) according to the manufacturer’s instructions. Fifty nanograms of total RNA was used for quantitating the SARS-CoV-2 genome. Viral RNA was quantified using a 1-step reverse transcription qRT-PCR, as described previously (23). Viral loads were calculated as copies per 1 g lung.

### Multiple cytokine expression analyses

The left lung lobes were lysed in lysis buffer (1% Triton-X100, 20 mM EDTA, 50 mM Tris-Cl pH 7.5, 150 mM NaCl) containing complete protease inhibitor (Sigma Aldrich, MO, USA), and were assayed using the Bio-plex Suspension Array System, which utilizes Luminex-based technology. A Mouse Cytokine/Chemokine Magnetic Bead Panel (32-plex) was used according to the manufacturer’s instructions (Merck KGaA, Darmstadt, Germany).

### Immunohistochemistry

The mice lungs were fixed in 10% neutral buffered formalin, embedded in paraffin, sectioned at a thickness of 4-μm, stained with HE, and subjected to routine histological examination. Paraffin block sections were also used for staining of the hACE2 and SARS-CoV-2 N protein. Antigen retrieval was performed by autoclaving sections in 10 mM citrate buffer (pH 6.0) for 10 min and then immersing the sections in 0.3% hydrogen peroxide in methanol at room temperature for 30 min to inactivate endogenous peroxidase. The sections were blocked with BlockAce (DS Pharma Biomedical, Osaka, Japan) for 15 min, and incubated overnight at 4°C with 0.2 μg/mL of rabbit anti-ACE2 polyclonal antibody [N1N2] (GenTex, Inc., CA, USA) or 2 μg/mL of rabbit anti-SARS-CoV-2 N protein monoclonal antibody [HL344] (GenTex). Secondary labeling was performed by incubation at RT for 30 min with EnVision+ System-HRP labeled Polymer Anti-Rabbit (Dako Denmark A/S, Glostrup, Denmark), followed by color development with ImmPACT DAB Peroxidase Substrate (Vector Laboratories, Burlingame, CA, USA) at RT for 10 min. Nuclear staining was performed with hematoxylin solution. For co-staining of the hACE2 and SARS-CoV-2 N protein, the sections were incubated overnight at 4°C with 0.2 μg/mL of rabbit anti-ACE2 polyclonal antibody [N1N2] (GenTex) and 1 μg/mL of rat anti-SARS-CoV-2 N protein monoclonal antibody [45C10-2] (unpublished data, material available upon request, used only in **Supplementary Figure 3**). Secondary labeling was performed by incubation at RT for 60 min with Goat Anti-Rabbit IgG H&L (Abcam, Cambridge, UK) and Goat Anti-Rat IgG H&L (HRP polymer) (Abcam), followed by color development with ImmPACT DAB Peroxidase Substrate (Vector Laboratories) and Vector Blue Substrate Kit, Alkaline Phosphatase (Vector Laboratories).

### Tandem mass tag pro 12-plex mass spectrometry analysis

Lysates extracted from left lung lobes were processed and digested by using an EasyPep Mini MS Sample Prep kit (Thermo Fisher Scientific) according to the manufacturer’s protocol. Three mouse lungs were pooled, and 25 µg of peptides from each sample were labeled with 0.25 mg of TMTpro mass tag labeling reagent (Thermo Fisher Scientific) according to the manufacturer’s protocol. After TMT labeling, the 8 sample channels were combined in equal proportions, dried using a speed-vac, and resuspended in 0.1% TFA. Samples were fractionated into 8 fractions using a High pH Reversed-Phase Peptide Fractionation Kit (Thermo Fisher Scientific) according to the manufacturer’s protocol. One microgram of peptide from each fraction was analyzed by LC-MS/MS on an EASY-nLC 1200-connected Orbitrap Fusion Lumos Tribrid mass spectrometer (Thermo Fisher Scientific) equipped with an FAIMS-Pro ion mobility interface (Thermo Fisher Scientific). Peptides were separated on an analytical column (C18, 1.6 µm particle size × 75 µm diameter × 250 mm, Ion Opticks) using 4-hr gradients (0% to 28% acetonitrile over 240 min) with a constant flow of 300 nL/min. Peptide ionization was performed using a Nanospray Flex Ion Source (Thermo Fisher Scientific). FAIMS-Pro was set to three phases (-40, -60, and -80 CV) and a ‘1 sec cycle for a phase’ data-dependent acquisition method was used where the most intense ions in every 1 sec were selected for MS/MS fragmentation by HCD. MS raw files were analyzed using a Sequest HT search program in Proteome Discoverer 2.4 (Thermo Fisher Scientific). MS/MS spectra were searched against the SwissProt reviewed mouse reference proteome (UniProt). TMTpro-based protein quantification was performed using the Reporter Ions Quantifier node in Proteome Discoverer 2.4.

### Statistical analysis

Statistical analyses were performed with Prism software (version 9.1.2; GraphPad, San Diego, CA, USA). Statistical significance was assigned when p values were <0.05. Inferential statistical analysis was performed by one-way analysis of variance (ANOVA), followed by Tukey's test or Mann–Whitney *U* test, as appropriate.

## Results

### Development of a severe pneumonia mouse model for SARS-CoV-2 infection using an hACE2 expressing adenoviral vector

**Figure 1:**
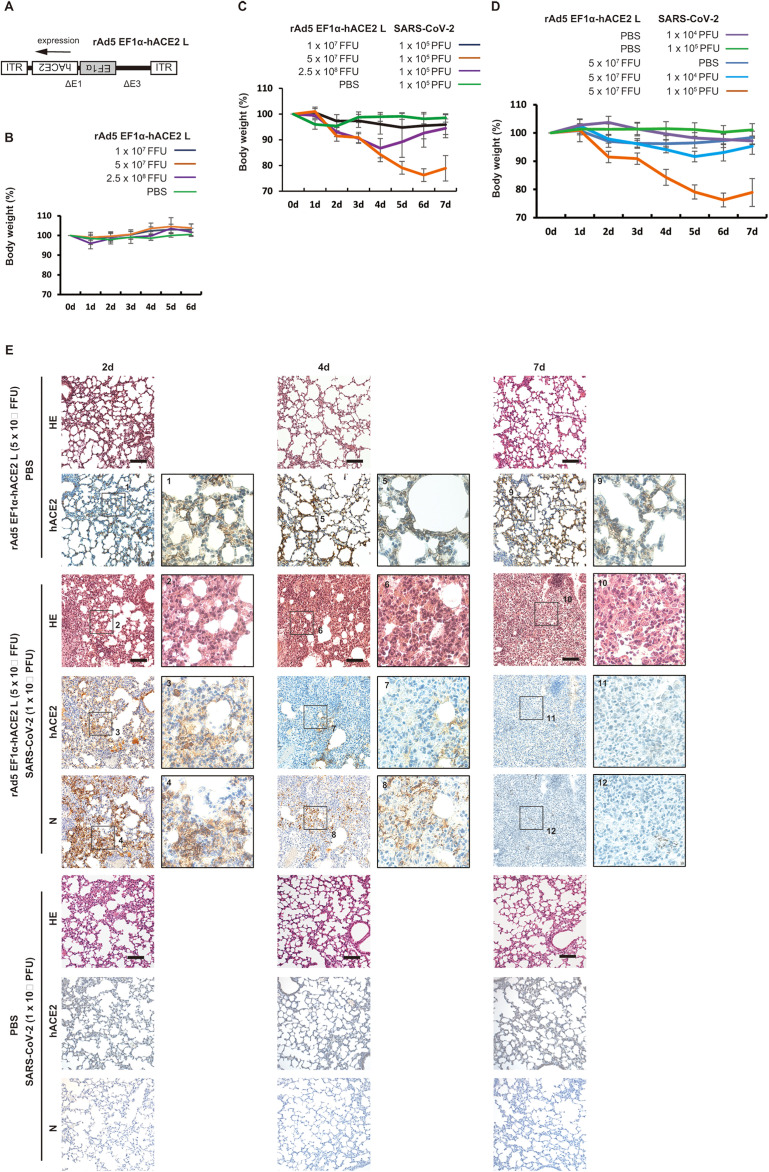
Development of a severe pneumonia mouse model following SARS-CoV-2 infection transduced with rAd5 pEF1α-hACE2-L. **(A)** Schematic diagram of rAd5 pEF1α-hACE2-L. ITR indicates an inverted terminal repeat. Δ E1 and Δ E3 indicate the deletion of E1 and E3 genes from the adenoviral genome. **(B)** Changes in body weight of rAd5 pEF1α-hACE2-L-inoculated BALB/c mice. Data represent means and standard deviations (SD), *n*=3 (6d), 6 (5d), 9 (4d), 12 (3d), 15 (2d), and 18 (0d and 1d). **(C)** Changes in body weight of BALB/c mice transduced with rAd5 pEF1α-hACE2-L (or PBS control), followed by infection with SARS-CoV-2 (Wu-2020). Mice were first inoculated with rAd5 pEF1α-hACE2-L at doses of 1×10^7^, 5×10^7^ or 2.5×10^8^ FFU/animal, and 5 dpi, SARS-CoV-2 Wu-2020 was administrated at 1×10^5^ PFU/animal. Day 0 equals 5 dpi of rAd5 pEF1α-hACE2-L administration. Data represent means and SD, *n*=6. **(D)** Changes in body weight of BALB/c mice transduced with rAd5 pEF1α-hACE2-L (5 × 10^7^ FFU/animal) or PBS control followed by infection with SARS-CoV-2 Wu-2020 at different doses. Day 0 equals 5 dpi of rAd5 pEF1α-hACE2-L. Mock indicates mice transduced with rAd5 pEF1α-hACE2-L, but not infected with SARS-CoV-2. Data represent means and SD, *n*=6. **(E)** Histopathological analyses of mouse lungs with Hematoxylin-Eosin (HE) staining and immunohistochemistry using antibody against hACE2 and SARS-CoV-2 N. The images depict one representative from six mice. Scale bars represent 100 µm.

We generated an adenoviral vector expressing hACE2 under the EF1α promoter with a leftward orientation (rAd5 pEF1α-hACE2-L) (**Figure 1A**). We first examined whether intranasal administration of rAd5 pEF1α-hACE2-L affects the body weight of BALB/c mice, and found that administration at 1 × 10^7^, 5 × 10^7^ or 2.5 × 10^8^ FFU per mouse did not cause any decrease in body weight when they were compared with those inoculated with PBS (**Figure 1B**). The expression of hACE2 in the lungs of mice inoculated with rAd5 pEF1α-hACE2-L was examined immunohistochemically using antibodies against hACE2 (**Supplementary Figure 1**). In both 1 × 10^7^ and 5 × 10^7^ FFU administration groups, hACE2 expression was observed in alveolar macrophages at 1 to 3 days post infection (dpi), and in the type I and II alveolar epithelial cells thereafter. This expansion of hACE2 expression was seen rapidly in the 5 × 10^7^ FFU group, with hACE2 expression observed throughout the tissue at 5 dpi and maintained until 6 dpi. In the 2.5 × 10^8^ FFU group, hACE2 expression was observed in both alveolar macrophages and epithelial cells, even at 1 dpi, and was maintained up to 6 dpi. However, marked simultaneous thickening of the alveolar wall was observed in this administration group.

Next, BALB/c mice were intranasally administered with rAd5 pEF1α-hACE2-L at 1 × 10^7^, 5 × 10^7^ or 2.5 × 10^8^ FFU/animal. The PBS administered group was used as a control. Five days after administration, mice were further intranasally inoculated with an early circulating strain of SARS-CoV-2 isolated in Japan (originally from Wuhan; Wu-2020 strain) at 1 × 10^5^ plaque forming units (PFU) per mouse (**Figure 1C**). As a result, the 5 × 10^7^ FFU rAd5/mouse group showed symptoms which manifested as ruffled fur, reduced activity, and marked reduction in body weight, which peaked at 5-6 dpi with SARS-CoV-2 Wu-2020. Although the 2.5 × 10^8^ FFU rAd5/mouse group showed weight loss until 4 dpi of SARS-CoV-2 infection, this group showed recovery of body weight after 5 dpi. The thickening of the alveolar wall was remarkable in the 2.5 × 10^8^ FFU/animal administered group at 5-6 dpi (**Supplementary Figure 1**), suggesting that the cytotoxicity induced by high-titer adenoviral infection may have resulted in the suppression of the SARS-CoV-2 infection and propagation. The 1 × 10^7^ FFU rAd5/mouse group showed a slight reduction of weight followed by rapid recovery. Therefore, we determined that 5 × 10^7^ FFU rAd5/animal was the most suitable dose for assessing disease severity. We next examined the body weight of mice inoculated with rAd5 (5 × 10^7^ FFU rAd5/animal) or PBS followed by infection with SARS-CoV-2 Wu-2020 at 1 × 10^4^ and 1 × 10^5^ PFU/mouse. Comparison of low (1 × 10^4^ PFU/mouse) and high (1 × 10^5^ PFU/mouse) titers of SARS-CoV-2 infection demonstrated that weight loss was more apparent in the high-titer group, with a maximum of approximately 25% (**Figure 1D**). Dark red and brown lesions were observed on the surfaces of lungs infected with 1×10^5^ PFU of SARS-CoV-2 at 7 dpi (**Supplementary Figure 2A**). Histopathologically, SARS-CoV-2 infected lungs (1 × 10^5^ PFU/mouse) showed severe pneumonia characterized initially by thickened epithelium (2 dpi) and further with profound infiltration of leukocytes, including macrophages, neutrophils, and lymphocytes, hemorrhaging, and increased blood coagulation (4 to 7 dpi) (**Figure 1E**, **Supplementary Figure 5**). These pathological findings were not observed in the PBS treated group instead of rAd5 administration. The expression of SARS-CoV-2 nucleoprotein (N) was evident on alveolar epithelial cells at 2 dpi, but attenuated from 4 to 7 dpi (**Figure 1E**). There were no signals for SARS-CoV-2 N and hACE2 in the lungs of mice inoculated with PBS instead of rAd5 administration. In the mock group (rAd5 administered but SARS-CoV-2 non-infected group), hACE2 expression in the alveolar epithelial cells persisted up to 7 dpi, but in the SARS-CoV-2 infected group, the hACE2-expressing cells disappeared at 4 dpi and were almost completely depleted at 7 dpi (**Figure 1E**). Co-immunostaining revealed the co-localization of hACE2 and SARS-CoV-2 N in alveolar epithelium and macrophages at 2 dpi with SARS-CoV-2, indicating that SARS-CoV-2 infection is dependent on the exogenous expression of hACE2 by rAd5 pEF1α-hACE2-L (**Supplementary Figure 3**, Wu-2020).

### Comparison of pathogenesis of SARS-CoV-2 variants in the mouse model

**Figure 2:**
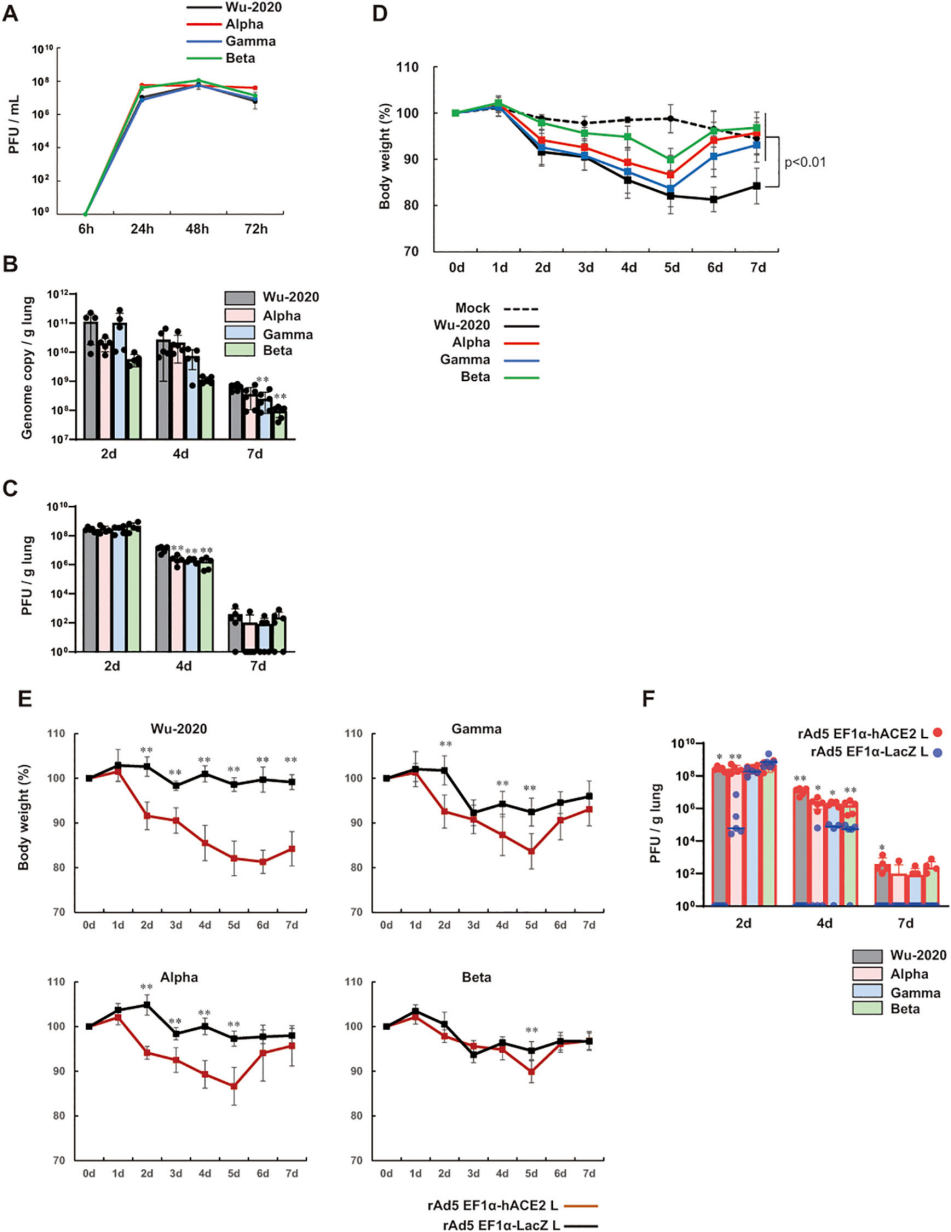
Comparison of pathogenesis of SARS-CoV-2 variants in the mouse model **(A)** Viral growth in Vero E6/TMPRSS2 cells at an MOI of 0.001. Viral titers are shown in PFU/mL, which were calculated by a standard plaque assay using Vero E6/TMPRSS2 cells. Data represent means and SD, *n*=3. **(B)** Viral loads in lung homogenate determined by qRT-PCR to detect the SARS-CoV-2 genome. **(C)** Viral titers in lung homogenate determined by a plaque assay using Vero E6/TMPRSS2 cells. Data represent means and SD, *n*=5 (2d and 4d) and *n*=6 (7d). **p<0.01 (one-way ANOVA followed by Tukey’s test, vs Wu-2020). **(D)** Changes in body weight of BALB/c mice transduced with rAd5 pEF1α-hACE2-L followed by infection of SARS-CoV-2 variants. Day 0 equals 5 dpi of rAd5 pEF1α-hACE2-L (5 × 10^7^ FFU/animal). The p value is shown (Wu-2020 vs mock and all variants) (one-way ANOVA followed by Tukey’s test). Data represent means and SD, *n*=6. **(E)** Changes in body weight of SARS-CoV-2 variant-infected BALB/c mice. Day 0 equals 5 dpi of rAd5 pEF1α-hACE2-L or rAd5 pEF1α-LacZ-L (5 × 10^7^ FFU/animal). Data represent means and SD, *n*=5. **p<0.01 (Mann–Whitney *U* test). **(F)** Viral titers in lung homogenate determined by a plaque assay using Vero E6/TMPRSS2 cells. Data represent means and SD, *n*=5. *p<0.05, **p<0.01 (Mann–Whitney *U* test).

Variants of concern such as Alpha, Beta, and Gamma have emerged showing evidence of altered virus characteristics (24). These variants have been associated with increased transmissibility, evasion of immunity from infection and vaccination, and reduced susceptibility to antibody therapies (25–27). Large population studies have observed a significant trend toward increased mortality associated with B.1.1.7 (Alpha) (28, 29). We studied the pathogenesis of variants [QHN001 (B.1.1.7, Alpha), TY7-501 (P.1, Gamma) and TY8-612 (B.1.351, Beta)], which were clinically isolated in Tokyo in 2021. We first examined the growth characteristics of these variants and the Wu-2020 strain used in this study in Vero E6/TMPRSS2 cells (18) at a multiplicity of infection (MOI) of 0.001, and found that the growth kinetics were almost comparable among strains (**Figure 2A**). To analyze their replication in mouse lungs, we inoculated the rAd5 pEF1α-hACE2-L administered BALB/c mice with SARS-CoV-2 via the intranasal route. Viral replication in the lungs was examined by quantitative real-time RT-PCR (qRT-PCR) for the detection of SARS-CoV-2 genome, and by plaque assay using Vero E6/TMPRSS2 cells. A clear increase in viral replication was observed with a peak at 2 dpi in all strains (**Figures 2B, C**), followed by a gradual decrease towards 7 dpi. The Gamma and Beta strains showed relatively reduced genome copy numbers (significantly at 7 dpi), but other strains showed comparable genome copy numbers throughout the course of infection. In Wu-2020- and Alpha strain-infected lungs, multiple dark red and brown lesions appeared on the surfaces of all lung lobes from 4 to 7 dpi (**Supplementary Figure 2B**, arrows). In contrast, the discolored lesions were restricted to the upper left lobe in Gamma and Beta strain-infected lungs at 7 dpi. Mice inoculated with all strains of SARS-CoV-2 exhibited symptoms of ruffled fur, reduced activity and loss of body weight. The loss of body weight in mice did not correspond to the extent of viral replication and lung lesions (**Figure 2D**). Although viral replication was similar in the Alpha and Gamma strains, the appearance of lung lesions was more pronounced in Alpha, however, weight loss was relatively more pronounced in Gamma. Mice infected with all four strains continued to lose weight until 5 dpi, but mice infected with three variants began to recover thereafter. Though viral replication and the extent of lung lesions were comparable in Alpha- and Wu-2020-infected mice, mice infected with Wu-2020 did not regain weight, resulting in significant weight loss at 7 dpi compared with mice infected with other variants (**Figure 2D**). To examine whether viral replication and symptoms by infection with SARS-CoV-2 Wu-2020 and variants were dependent on the exogenously expressed hACE2, we prepared an adenovirus vector that expresses β-galactosidase (LacZ) instead of hACE2 (rAd5 pEF1α-LacZ-L) (22). BALB/c mice were administered with rAd5 pEF1α-LacZ-L at 5 × 10^7^ FFU/animal, and five days later, they were inoculated with SARS-CoV-2 Wu-2020, Alpha, Gamma, and Beta at 1 × 10^5^ PFU/animal. The Wu-2020- and Alpha-infected mice showed no weight loss, while the Gamma- and Beta-infected mice showed slight weight loss at 3 dpi, but recovered quickly (**Figure 2E**). Infectious SARS-CoV-2 was detected in the lungs of Alpha-, Gamma-, and Beta-infected mice at 2 dpi, but it eventually disappeared more rapidly than in animals inoculated with rAd5 pEF1α-hACE2-L (**Figure 2F**). At least Beta-strain seems to be able to infect mouse lungs with or without rAd5 pEF1α-hACE2-L inoculation (**Supplementary Figure 3**). Proliferation of these viruses in the lung did not appear to lead to the development of symptoms. SARS-CoV-2 Beta and Gamma can infect wild-type mice without exogenous expression of human ACE2 (30). However, pre-administration of rAd5 pEF1α-hACE2-L appears to more reliably enhance the efficiency of these SARS-CoV-2 infections.

**Figure 3:**
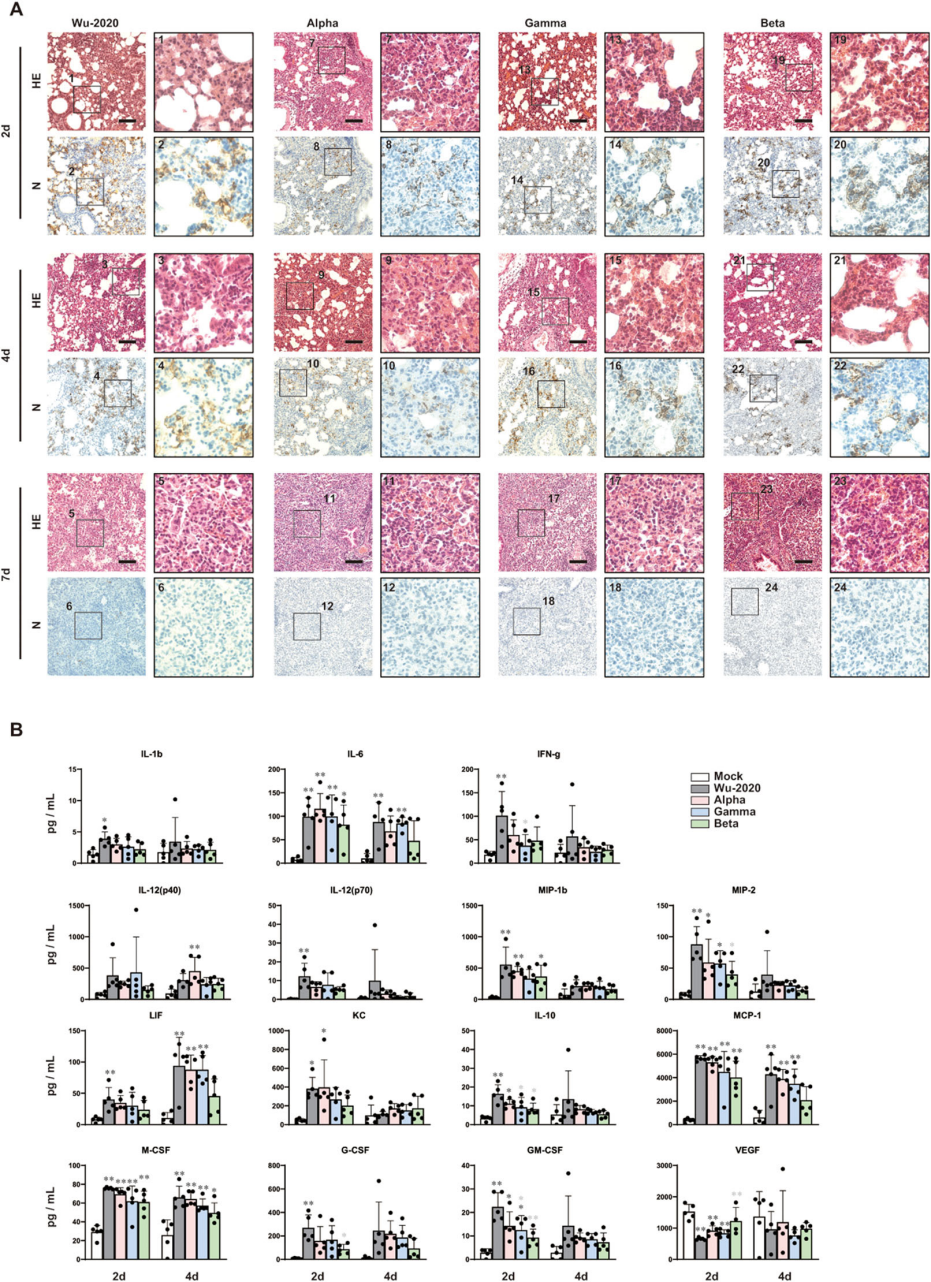
Histopathological analyses and cytokine levels of mouse lung infected with SARS-CoV-2 variants **(A)** Histopathologic findings with HE staining and detection of SARS-CoV-2 N protein in mouse lungs infected with SARS-CoV-2. The images depict one representative from five (2 d and 4 d) and six (7 d) mice. Scale bars represent 100 µm. **(B)** Left lung homogenates were used for measurement of multiplex cytokines and chemokines using the Bio-plex suspension array system. Data represent means and SD, *n*=5. *p<0.05, **p<0.01 (one-way ANOVA followed by Tukey’s test). The colors of the asterisks indicate the following: black (vs mock) and gray (vs Wu-2020).

Next, we performed histopathological analysis of Wu-2020, Alpha, Gamma, and Beta-infected lungs in rAd5 pEF1α-hACE2-L inoculated mice, and found little difference among these four strain-induced diseases (**Figure 3A**). In all SARS-CoV-2-infected lungs, thickening of the alveolar wall was observed at 2 dpi, where the SARS-CoV-2 N expression was specifically detected. The differences in the distribution of viral antigens and host cell specificity among virus strains were not clear. Viral antigens then began to diminish by 4 dpi and almost disappeared by 7 dpi in all virus strains. Increases in inflammatory cell infiltration, as well as hemorrhage and blood coagulation were observed in all strain-infected lungs at 4 and 7 dpi (**Figure 3A**, **Supplementary Figure 4**). Gamma and Beta infection seem to induce a slight inflammatory response without rAd5 pEF1α-hACE2-L administration.

### Cytokine expression in the SARS-CoV-2-infected lung

Next, we examined cytokine expression in the lung using a multiplex bead array. Inflammatory cytokines, such as IL-6, were significantly elevated in all strains compared to mock-infected animals (**Figure 3B**). Furthermore, IL-1b, IFN-g, IL-12, MIP-1b, MIP-2, LIF, KC, IL-10, MCP-1, M-CSF, G-CSF, and GM-CSF were significantly elevated and VEGF was decreased, at least in one strain compared to mock-infected mice. In Beta-infected lungs, the expression of some cytokines, such as MIP2, LIF, and MCP-1, was relatively low compared to other strains. For infection by other strains, there was no clear evidence of a relationship between cytokine levels and disease severity.

### Proteome analysis of SARS-CoV-2-infected lungs

**Figure 4:**
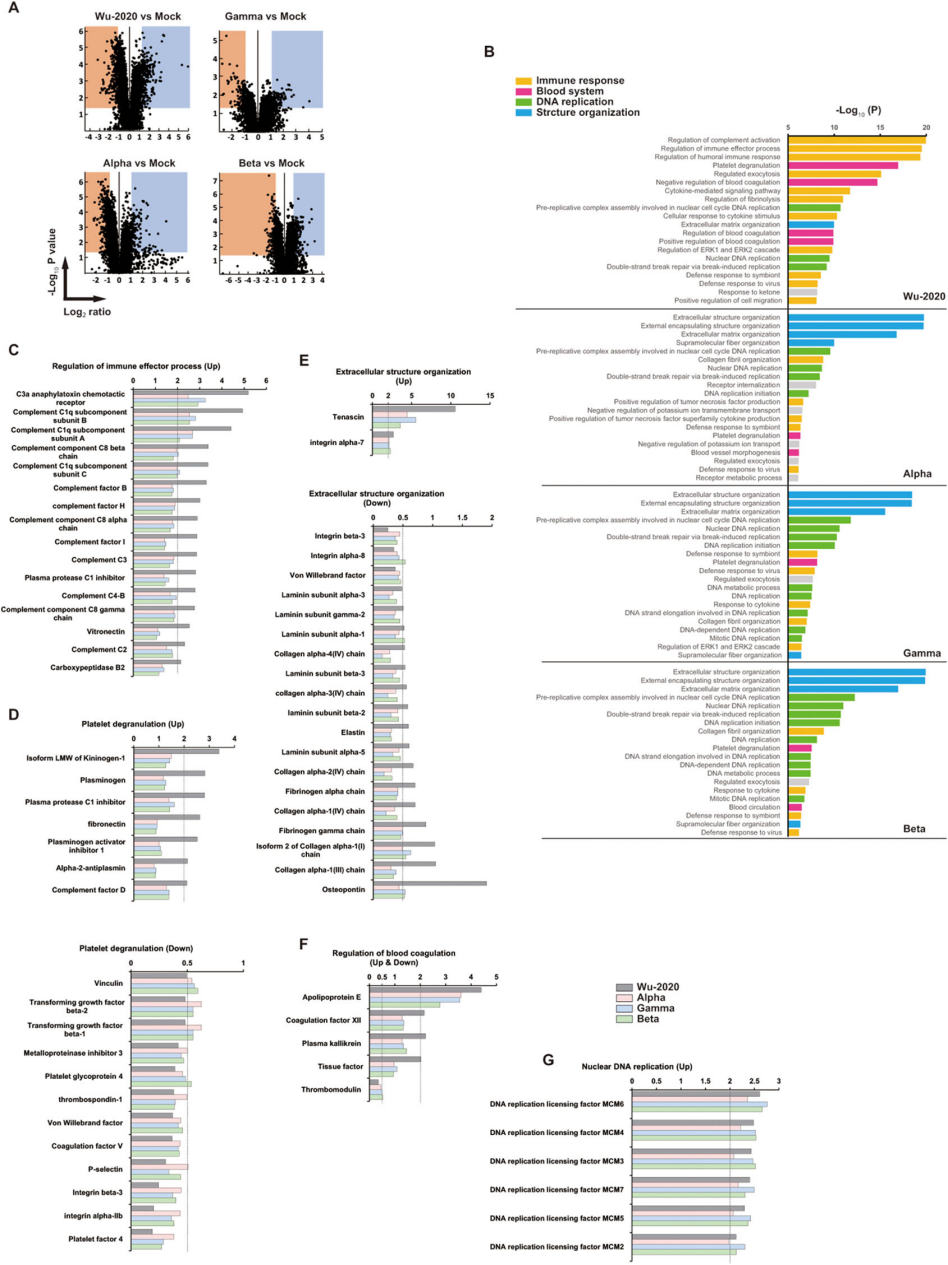
Proteomic landscape of SARS-CoV-2 infected mouse lungs **(A)** Volcano plots for mouse lung proteome of the indicated group compared to adenovirus-infected/SARS-CoV-2 non-infected (mock) mice. Up-regulated (Log_2_ ≥ 1) and down-regulated (Log_2_ ≤ -1) proteins and p value < 0.05 indicate threshold lines. The numbers of up- and down-regulated proteins are 403 and 411 (Wu-2020 vs Mock), 206 and 289 (Alpha vs Mock), 283 and 236 (Gamma vs Mock), 249 and 189 (Beta vs Mock). The lung lysates used for proteomics were generated by combining three mouse lung homogenates, and the two combined lysates per each group were used for analysis. **(B)** GO enrichment analysis in proteomes of SARS-CoV-2-infected mouse lungs. Top 20 terms of each group are shown. Terms were further categorized into immune response (orange), blood system (pink), DNA replication (green) and structure organization (blue). **(C-G)** Intrinsic relative protein expressions were categorized into regulation of immune effector process **(C)**, platelet degranulation **(D)**, extracellular structure organization **(E)**, regulation of blood coagulation **(F)** and nuclear DNA replication **(G)**. X axes indicate relative values shown as fold change when that of mock equals 1. The dashed lines represent the thresholds of up-regulation (>2) and down-regulation (<0.5).

The clinical severity of COVID-19 is not always associated with increased levels of pro-inflammatory cytokines and other inflammation markers (31). To survey the molecules associated with disease severity, TMT peptide labeling, combined with MS quantitative proteomics in mouse lung at 7 dpi with SARS-CoV-2 was performed. The TMT-based quantitative proteomic method was approved previously for comparison of protein levels across multiple organs in human COVID-19 autopsy cases (32). TMTpro 12-plex MS revealed distinct lung proteomes associated with infection by SARS-CoV-2 strains (**Figure 4A**, **Supplementary Figure 5A**). Gene ontology (GO) enrichment analysis of significantly (p<0.05) up- and down-regulated (2-fold) proteins showed that the proteome of Wu-2020-infected lung was distinct from those of other variants (**Figure 4B**). Immune response-related factors, such as regulation of complement activation, immune effector process, as well as platelet degranulation and regulation of blood coagulation, were enriched in the proteins that changed significantly in the proteome of Wu-2020-infected lungs (**Figure 4B**). In contrast, the proteomes of other variant-infected lungs were associated with structural organization, such as the development of extracellular structures and changes in matrix organization, as well as nuclear DNA replication. Up-regulation of proteins associated with complement activation, e.g., C3a anaphylatoxin chemotactic receptor, complement components and complement factors was prominent in Wu-2020-infected lungs (**Figure 4C**). The complement system has been shown to be involved in the severity of human COVID-19 (33, 34). Up-regulation of proteins involved in platelet degranulation and blood coagulation, e.g., kininogen, fibronectin, plasminogen activator inhibitor-1, coagulation factor XII and plasma kallikrein, was also remarkable in Wu-2020-infected lung tissue (**Figures 4D, F**). These factors are considered to work in concert and contribute to COVID-19 pneumonia via dysregulation of thrombus formation. Up-regulation of minichromosome maintenance complex component (MCM) 2, 3, 4, 5, 6, and 7, which are related to nuclear DNA replication, was observed in SARS-CoV-2 infection, regardless of strain (**Figure 4G**). MCM2-7 act as replicative DNA helicases that unwind the DNA duplex template as a hetero-hexameric complex (35). The involvement of the MCM family in immune responses against viral infection is still poorly characterized. However, MCM up-regulation is correlated with proliferation and maintenance of leukocytes (36, 37), suggesting that the MCM family is involved in the activation of infiltrating cells in the COVID-19 pneumonia observed in lungs infected with all of the virus strains. Structural organization-related proteins, such as collagens, were down-regulated in Alpha-, Beta-, and Gamma-infected lungs (**Figure 4E**). Collagen deposition is a hallmark of lung fibrosis (38) and has been confirmed in the lungs of COVID-19 patients (39). It is considered that collagen deposition may be correlated with mild disease onset based on the recovery of body weight in Alpha-, Beta-, and Gamma-infected mice. Pathway analysis based on Wikipathways (https://www.wikipathways.org/index.php/WikiPathways) supports the enrichment of complement and coagulation cascades, as well as the blood-clotting cascade, in Wu-2020-infected lungs (**Supplementary Figure 7**).

## Discussion

We established a system to recapitulate COVID-19-like pneumonia in mice infected with SARS-CoV-2 after inducing hACE2 with rAd5 pEF1α-hACE2-L. When rAd5 pEF1α-hACE2-L was used, there were few abnormalities in protein expression (**Supplementary Figure 5A**), suggesting that this adenoviral vector has low cytotoxicity. Mice infected with the Wu-2020 strain developed diffuse pneumonia. Histopathologically, thickened alveolar walls, hemorrhaging, and infiltration of inflammatory cells were prominent. The SARS-CoV-2 N antigen was found in alveolar epithelial cells early in the course of the disease, but decreased as the pneumonia progressed. These immunohistopathological findings appear to be similar to human COVID-19 autopsy cases in early 2020 (40–42).

Although the viral vector-mediated hACE2 delivery system has been reported to result in milder symptoms of SARS-CoV-2 infection compared to those of K18-hACE2 Tg mice (43), it also has the advantage of rapidly sensitizing various laboratory mouse strains, leading to a reduction in costly and time-consuming genetic manipulation procedures for transgenic animals. The present mouse model using rAd5 pEF1α-hACE2-L exhibited more severe weight loss and pulmonary pathogenesis after SARS-CoV-2 early circulating strain inoculation than the previously reported COVID-19 mouse model using CMV promoter-driven hACE2 expressing adenoviral or AAV vectors (15, 16, 44). Our model system has the potential to accelerate the pace of research into viral replication and pathogenicity *in vivo* and facilitate the development of vaccines and therapeutics. In fact, we have already used this mouse model to evaluate vaccine efficacy (45).

SARS-CoV-2 Wu-2020, an early circulating strain, appeared to be highly pathogenic in mouse lung. The Wu-2020, Alpha and Gamma strains had comparable replication potentials in both Vero E6/TMPRSS2 cells and mouse lung tissue (**Figures 2A-C**). These strains also induced a marked cytokine response (**Figure 3B**), and infection led to similar histopathological outcomes (**Figure 3A**, **Supplementary Figure 4**). However, the most notable difference between Wu-2020 and the other variants was revealed through proteomic analysis of lung tissue (**Figure 4**, **Supplementary Figures 5**, **6**). The analysis showed that proteins involved in the complement system were significantly elevated in Wu-2020 infections (**Figures 4B, C**). The release of proinflammatory complement peptides facilitates the recruitment of leukocytes to the lung and contributes to the assembly of terminal complexes that damage vascular endothelium (33, 34, 46). Increased levels of complement fragments is related to disease severity in COVID-19 patients (33, 47), suggesting their potential as markers for severe lung injury in Wu-2020 infections. The study also observed alterations in the blood coagulation system, indicated by the up-regulation of thrombosis-related proteins such as tissue factor (TF), coagulation factor XII, and plasma kallikrein (**Figure 4F**). TF activates the extrinsic coagulation pathway to generate thrombin in response to tissue injury and inflammation (48–51). Coagulation factor XII is activated by polyphosphates released from platelets, and initiates an intrinsic coagulation cascade (52–54), which is known to be involved in the disease onset of acute respiratory distress syndrome (55). Factor XII also activates plasma kallikrein, thereby increasing the formation of the proinflammatory peptide bradykinin (56). Notably, despite the absence of fibrin deposition in the histological analysis of Wu-2020-infected lungs, several inhibitory factors for complement activation, such as complement factor H and vitronectin (**Figure 4C**), as well as for coagulation, such as plasminogen, which promotes fibrinolysis (**Figure 4D**), were up-regulated. These factors may play a role in preventing excessive tissue injury. These findings suggest that molecular alterations in pneumonia lesions, which are not detectable through morphological observation alone, could contribute to the severe symptoms observed in Wu-2020 infections, such as sustained weight loss.

In this study, levels of pro-inflammatory cytokines could not be used as markers of disease severity. Rather, the proteome results showed that factors related to complement may be one of the key factors associated with COVID-19 severity. In addition, we observed 35-fold and 9-fold up-regulation of metallothionein-2 (Mt2) and Mt1, respectively, in Wu-2020-infected lung (**Supplementary Figure 6**). Mt1/2, which are potently induced by heavy metals, other sources of oxidative stress and cytokines, facilitate metal binding and detoxification (57). In response to GM-CSF, macrophages express Mts (Mt2 rather than Mt1), which are involved in antimicrobial responses and contribute to the production of reactive oxidative species (58). We observed a correlation between disease severity and Mt1/2 amount, suggesting that Mt1/2 may act as a marker for COVID-19 severity. Additionally, we identified other potential biomarkers that may be correlated with disease severity, including tenascin, membrane-spanning 4-domains subfamily A member 6C and stefin-1/3 (**Supplementary Figure 6**), whose associations with COVID-19 have not been studied to date. Furthermore, abundance of the SARS-CoV-2 N protein in lungs infected with Wu-2020 was markedly higher than that in other strains (**Supplementary Figure 6**). SARS-CoV-2 N protein has been shown to promote NLRP3 inflammasome activation (59), and it is possible that SARS-CoV-2 N protein remaining in the lung may stimulate excessive inflammation. The amount of residual SARS-CoV-2 N protein in lesions may also be indicative of lung injury. It is possible that a comparison of SARS-CoV-2 strains that exhibit different pathogenicity may reveal the existence of novel biomarkers for disease severity.

Intrinsic etiological differences in Alpha, Gamma, and Beta have not yet been demonstrated. Although the Alpha, Gamma, and Beta strains do not show increased pathogenicity in hamsters (60–63) or rhesus macaques (64), Alpha and Beta strains showed high pathogenicity in the K-18 hACE2 Tg mouse model (65, 66). SARS-CoV-2 infection involves extra-respiratory manifestations, including cardiac, gastrointestinal, hepatic, renal, and neurological symptoms. Disease severity in K-18 mice infected with Alpha and Beta strains may be due to these extra-respiratory symptoms, as shown by the presence of neurological pathogenesis (67, 68). The transduction of mouse lungs with rAd5 pEF1α-hACE2-L only induces temporary hACE2 expression in lung but not other extrapulmonary organs, which explains the differences in the pathology of Wu-2020 and other variants whose pathology differs from that observed in other animal experimental models. The enhanced expression of hACE2 in the respiratory region may also prevent SARS-CoV-2, especially those variants with enhanced affinity toward hACE2 to colonize and replicate efficiently in the lower respiratory tract, which is another reason why we did not observe Alpha, Gamma, and Beta exhibiting higher pathogenicity than the Wu-2020.

To fully understand the evolving pathogenesis of SARS-CoV-2, it is important to extend these studies to include more recent variants, such as Omicron. The Omicron variant, known for its high transmissibility and extensive mutations, possesses unique characteristics that differ significantly from earlier strains. Comparative studies involving Omicron will help determine if the pathogenesis observed in early strains and variants like Alpha, Beta, and Gamma are consistent with those of Omicron. Investigating the proteomic changes induced by Omicron infection will provide critical insights into how this variant interacts with host tissues and contributes to disease severity.

## Data Availability

The datasets presented in this study can be found in online repositories. The names of the repository/repositories and accession number(s) can be found below: PDX046870 (PRIDE).

## Supplementary material

Supplementary Figure 1 The hACE2 expression in the mouse lung inoculated with rAd5 pEF1α-hACE2-L. Histopathologic findings with HE staining and detection of hACE2 in mouse lung inoculated with rAd5 pEF1α-hACE2-L at 1 × 10^7^, 5 × 10^7^ and 2.5 × 10^8^ FFU/animal for 1, 3, 5 and 6 dpi. The images depict one representative from three mice. Scale bars represent 100 µm.

Supplementary Figure 2 Macroscopic appearance of SARS-CoV-2-infected mouse lungs. **(A)** Macroscopic appearances of lungs of BALB/c mice transduced with rAd5 pEF1α-hACE2-L (5 × 10^7^ FFU/animal) followed by infection with SARS-CoV-2 Wu-2020 at different doses, at 7d postinfection with SARS-CoV-2. **(B)** Macroscopic appearance of lungs of BALB/c mice transduced with rAd5 pEF1α-hACE2-L (5 × 10^7^ FFU/animal) followed by infection with SARS-CoV-2 variants (1 × 10^5^ PFU/animal), at 2, 4, and 7d postinfection of SARS-CoV-2. Yellow arrows indicate lesions with a dark red and brown color. Mock indicates mice transduced with rAd5 pEF1α-hACE2-L, but not infected with SARS-CoV-2.

Supplementary Figure 3 Co-staining of hACE2 and SARS-CoV-2 N protein in the mouse lung inoculated with rAd5 pEF1α-hACE2-L and SARS-CoV-2 Wu-2020 or Beta. Histopathological findings of hACE2 and SARS-CoV-2 N co-staining in lungs of mouse inoculated with rAd5 pEF1α-hACE2-L (5 × 10^7^ FFU/animal) and SARS-CoV-2 Wu-2020 or Beta (1 × 10^5^ PFU/animal, 2 dpi). The images depict one representative from five mice. The scale bar represents 20 µm.

Supplementary Figure 4 Histopathological analysis of mouse lung infected with SARS-CoV-2 variants at 7 dpi. Histopathological findings of mouse lungs infected with SARS-CoV-2 variants by HE staining. **(A)** Overall view. **(B)** Magnified image showing thickening of the alveolar wall and infiltration of inflammatory cells. **(C)** Magnified image with prominent blood coagulation. The images depict one representative from six mice. Scale bars represent 100 µm.

Supplementary Figure 5 Proteomic landscape of SARS-CoV-2-infected mouse lungs compared with adenovirus non-infected/SARS-CoV-2 non-infected lungs **(A)** Volcano plots for mouse lung proteome of indicated group compared with adenovirus non-infected/SARS-CoV-2 non-infected (normal) lungs. Up-regulated (Log_2_ ≥1) and down-regulated (Log_2_ ≤ -1) proteins and p value < 0.05 indicate threshold lines. The numbers of up- and down-regulated proteins are 79 and 8 (Adeno vs normal), 591 and 410 (Wu-2020 vs normal), 405 and 277 (Alpha vs normal), 500 and 227 (Gamma vs normal), 468 and 146 (Beta vs normal). **(B)** The selected protein map shows the top 20 up- and down-regulated proteins in each category of the indicated virus-infected mouse lung proteome. Categories were determined based on GO terms; biological process 1.

Supplementary Figure 6 Top ten up-regulated proteins in SARS-CoV-2-infected mouse lungs. Intrinsic relative protein expression of SARS-CoV-2-infected lungs compared with adenovirus-infected/SARS-CoV-2 non-infected (mock) lungs. Y axes indicate relative values shown as fold change when that of mock equals 1.

Supplementary Figure 7 Pathway analysis in proteomes of mouse lungs infected with SARS-CoV-2 variants. Pathway maps in Wikipathways of up- and down-regulated proteins in mouse lung infected with indicated strains compared adenovirus-infected and non-SARS-CoV-2-infected (mock) mice. Top 20 pathways in each mouse proteome are shown.
