# Supplementary material for: Generation of a SARS-CoV-2-susceptible mouse model using adenovirus vector expressing human angiotensin-converting enzyme 2 driven by an elongation factor 1α promoter with leftward orientation
Source: Front Immunol. 2024 Dec 9;15:1440314. doi: 10.3389/fimmu.2024.1440314 (PMC11663739; doi:10.3389/fimmu.2024.1440314)
Supplement: Supplementary Figure 1 — The hACE2 expression in the mouse lung inoculated with rAd5 pEF1α-hACE2-L. Histopathologic findings with HE staining and detection of hACE2 in mouse lung inoculated with rAd5 pEF1α-hACE2-L at 1 × 10^7^, 5 × 10^7^ and 2.5 × 10^8^ FFU/animal for 1, 3, 5 and 6 dpi. The images depict one representative from three mice. Scale bars represent 100 µm. [file DataSheet1.pdf]

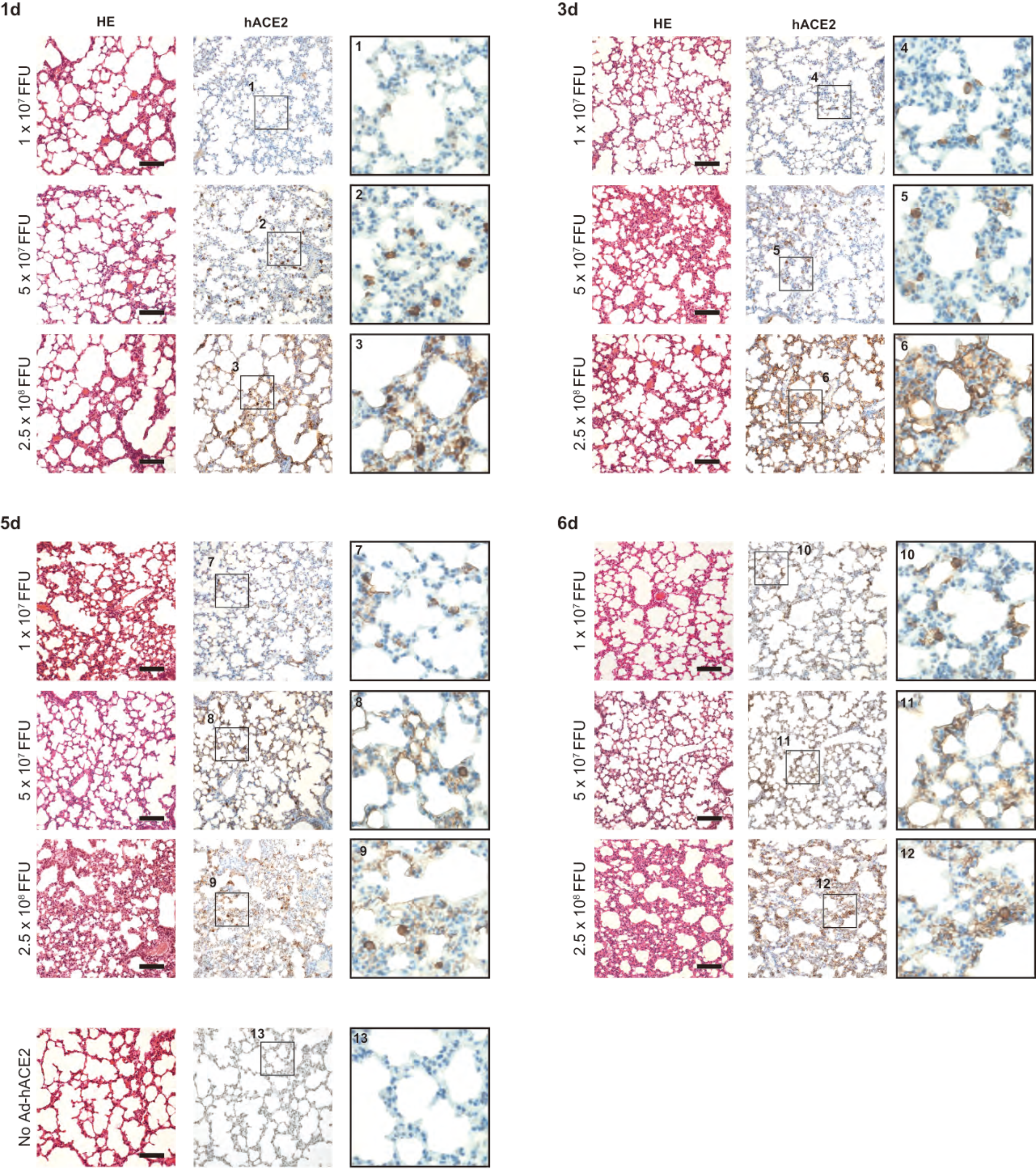

Supplementary Fig. 1

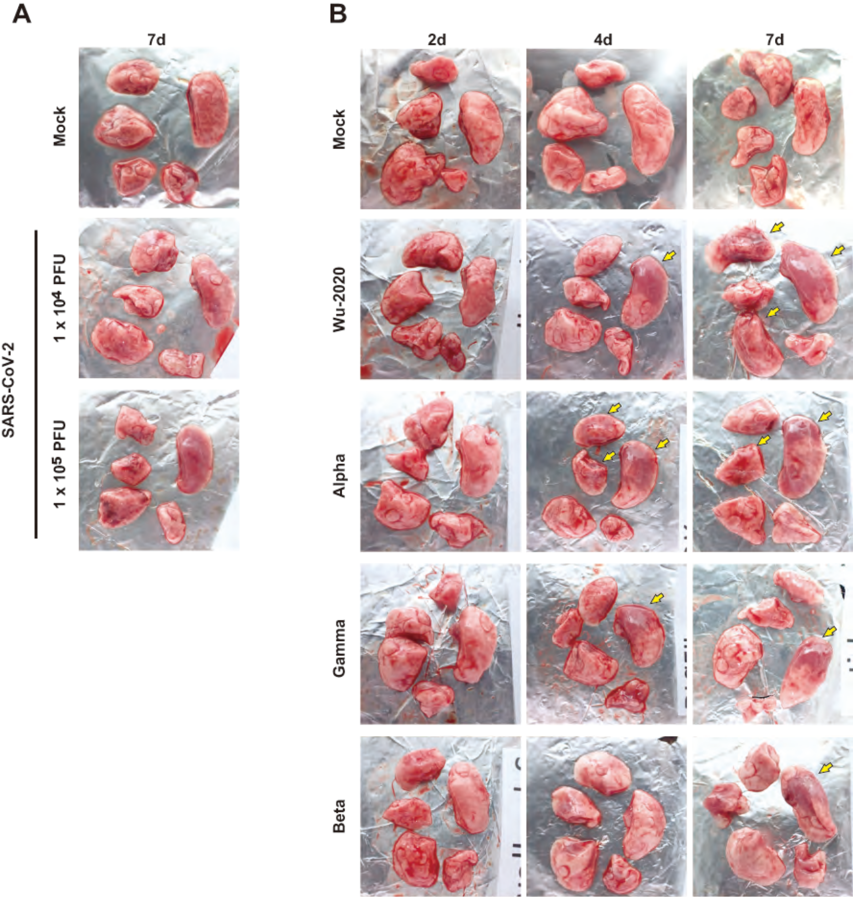

Supplementary Fig. 2

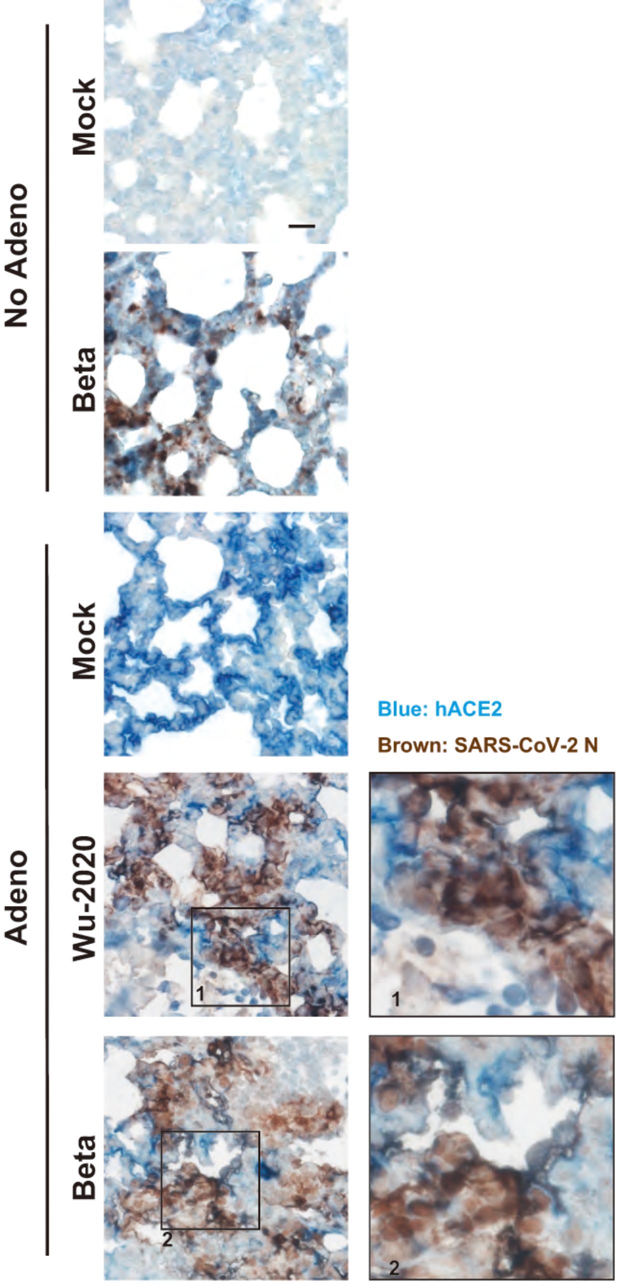

Supplementary Fig. 3

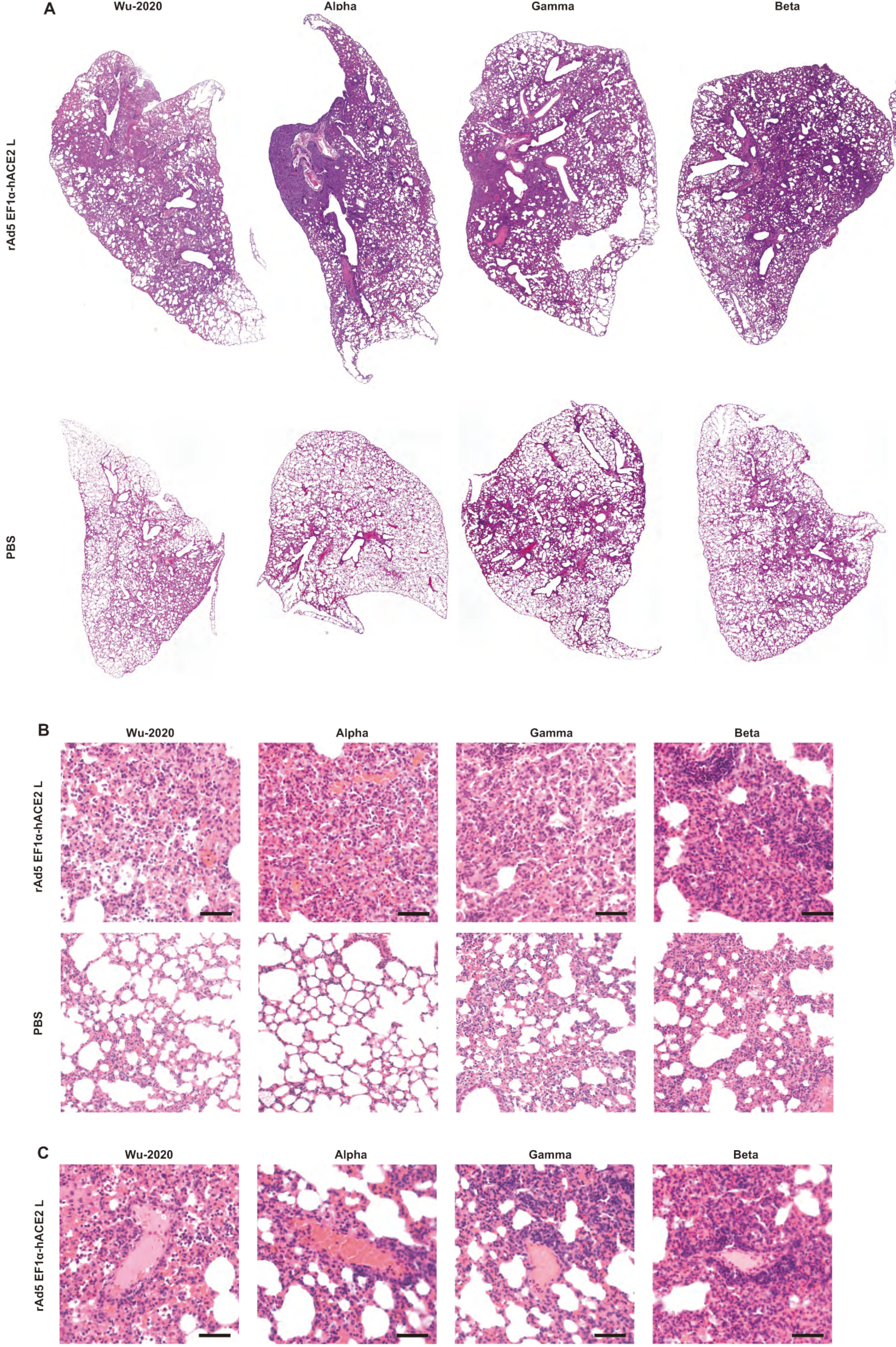

Supplementary Fig. 4

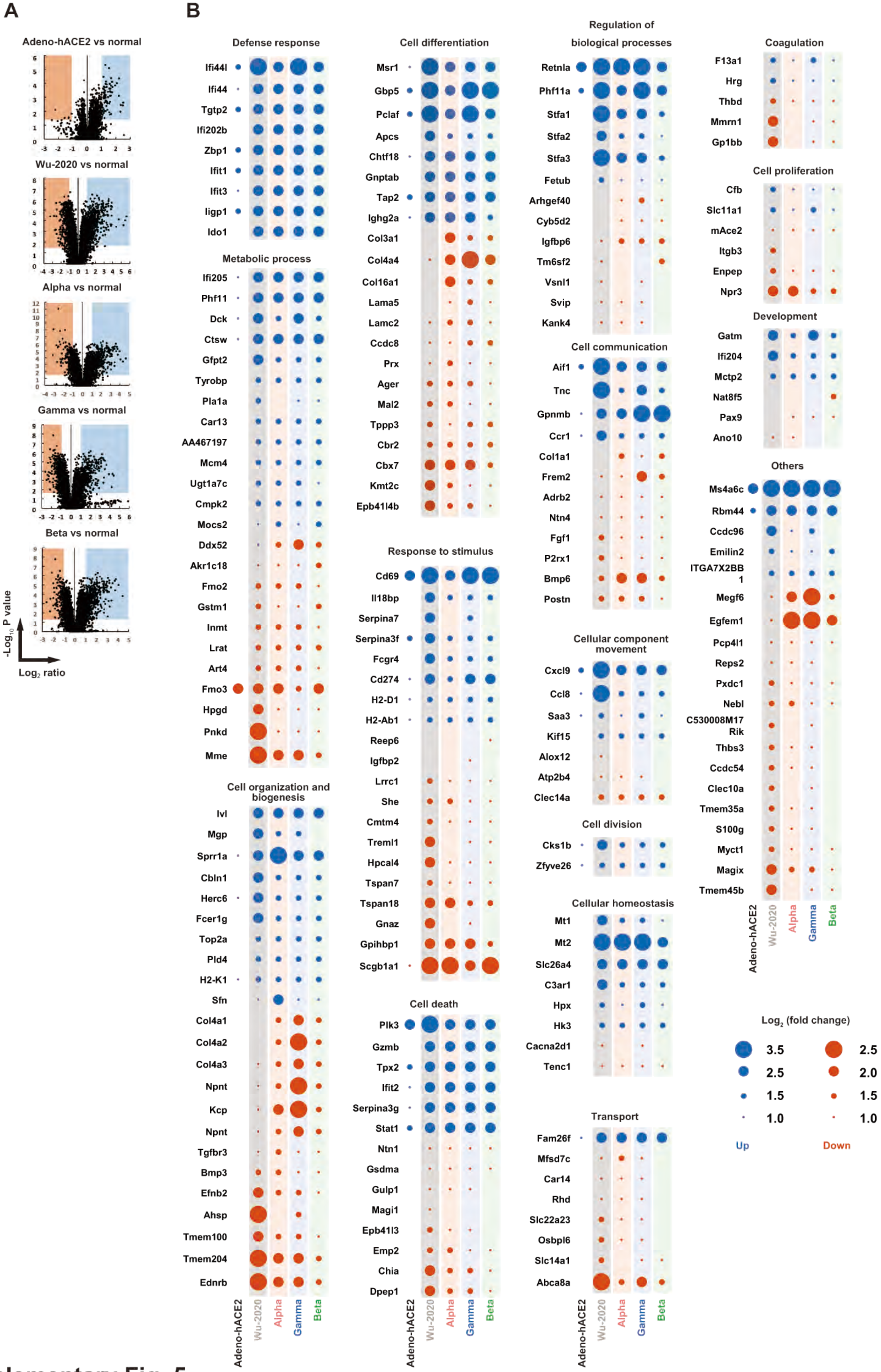

Supplementary Fig. 5

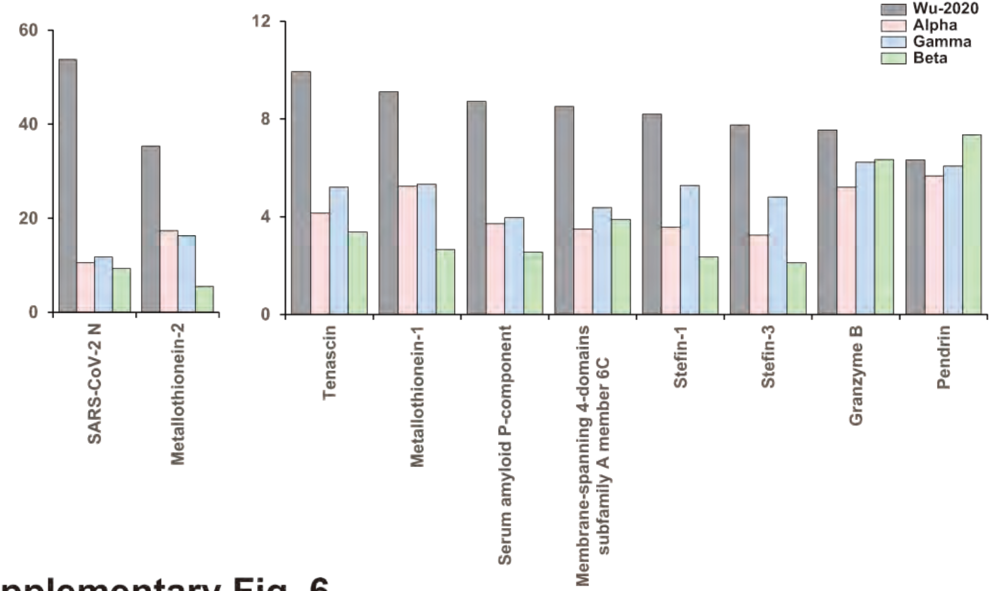

Supplementary Fig. 6

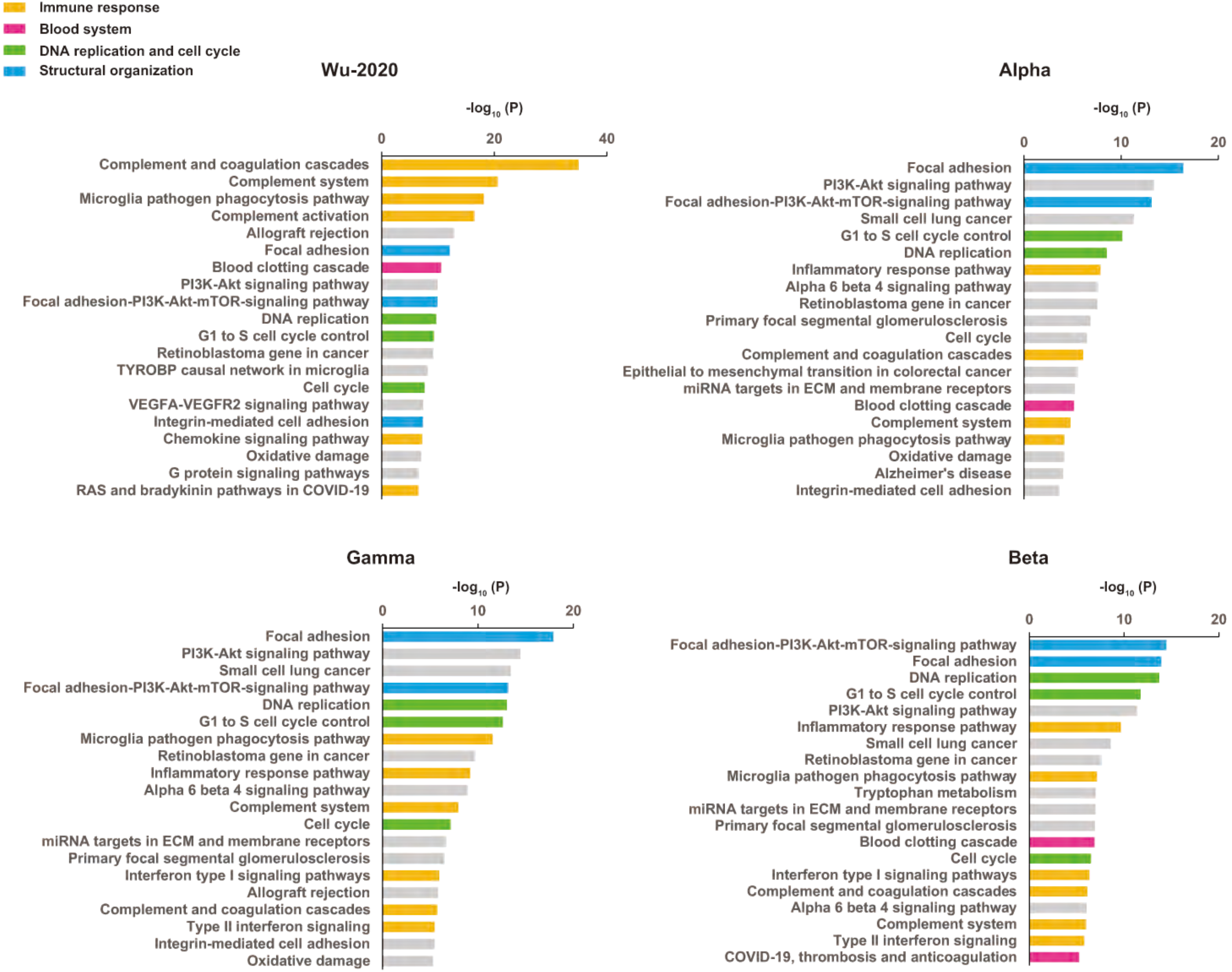

Supplementary Fig. 7
